# A Comparison of Norepinephrine versus Phenylephrine to Prevent Hypotension after Spinal Anesthesia for Cesarean Section: Systematic Review and Meta-Analysis

**DOI:** 10.3390/jpm14080803

**Published:** 2024-07-29

**Authors:** Hyun Kang, Tae-Yun Sung, Young Seok Jee, Woojin Kwon, Sung-Ae Cho, Somin Ahn, Choon-Kyu Cho

**Affiliations:** 1 Department of Anesthesiology and Pain Medicine, Chung-Ang University College of Medicine, Seoul 06911, Republic of Korea; roman00@naver.com; 2 Department of Anesthesiology and Pain Medicine, Konyang University Hospital, Konyang University College of Medicine, Daejeon 35365, Republic of Korea; sty200359@kyuh.ac.kr (T.-Y.S.); 200723@kyuh.ac.kr (W.K.); 200685@kyuh.ac.kr (S.-A.C.); 400957@kyuh.ac.kr (S.A.); kl6482@kyuh.ac.kr (C.-K.C.); 3 Myunggok Medical Research Institute, Konyang University Hospital, Konyang University College of Medicine, Daejeon 35365, Republic of Korea

**Keywords:** anesthesia, spinal, cesarean section, meta-analysis, norepinephrine, phenylephrine, hypotension, vasoconstrictor agents

## Abstract

Background: This systematic review and meta-analysis aimed to compare the effects of using phenylephrine or norepinephrine on the pH and base excess (BE) of the umbilical artery and vein in parturients undergoing cesarean section. Methods: The study protocol was registered in INPLASY. Independent researchers searched Ovid-Medline, Ovid-EMBASE, and Cochrane Central Register of Controlled Trials (CENTRAL) databases and Google Scholar for relevant randomized controlled trials (RCTs). The primary outcome of this study was the umbilical artery (UA) or umbilical vein (UV) pH as neonatal condition at birth, and the secondary outcome was the UA or UV BE as an additional prognostic value over the measurement of umbilical pH. Results: There was no evidence of a difference between phenylephrine and norepinephrine for overall, UA, and UV pH (mean difference (MD) −0.001, 95% confidence interval (CI) −0.004 to 0.007; MD 0.000, 95%CI −0.004 to 0.004; and MD 0.002, 95%CI −0.013 to 0.017). There was also no evidence of a difference between phenylephrine and norepinephrine for overall, UA, and UV BE (MD 0.096, 95% CI −0.258 to 0.451; MD 0.076, 95%CI −0.141 to 0.294; and MD 0.121, 95%CI; −0.569 to 0.811). A meta-regression showed that factors such as umbilical artery or vein, infusion method, single or twin, and the number of parturients per study had no effect on the UA pH, UV pH, UA BE, or UV BE. No evidence of publication bias was detected. Conclusions: There was no evidence of a difference between phenylephrine and norepinephrine for umbilical pH and BE. A subgroup analysis and meta-regression also did not show evidence of differences.

## 1. Introduction

The prevention and treatment of hypotension during spinal anesthesia for cesarean section is a major concern in the field of obstetric anesthesia. The use of vasopressors is the most reliable method for preventing and treating hypotension after spinal anesthesia for cesarean delivery.

In the past, ephedrine was the drug of choice because it maintains uterine blood flow through its adrenergic beta-effect [1]. After reports that ephedrine could lead to an acid–base imbalance in the fetus, it has now been replaced by phenylephrine [2]. However, phenylephrine can cause bradycardia and increase overall vascular resistance, leading to reduced maternal cardiac output. Glycopyrrolate is recommended to address the bradycardia induced by phenylephrine, but its effects are temporary and sometimes ineffective [3].

To overcome these problems, norepinephrine is being tried as a new alternative [4]. However, its safety for the fetus has not been conclusively established, prompting clinical studies that compare norepinephrine with phenylephrine in this context.

Therefore, the aim for this systematic review and meta-analysis was to compare the pH and BE of the umbilical artery and vein in parturients undergoing cesarean section when using phenylephrine and norepinephrine as vasopressor.

## 2. Materials and Methods

We developed the protocol for this systematic review and meta-analysis according to the Preferred Reporting Items for Systematic Review and Meta-Analysis Protocol (PRISMA-P) [5] and registered it in INPLASY (INPLASY202380048) on 12 August 2023.

This systematic review and meta-analysis was conducted while observing the recommendations by the Cochrane Collaboration [6] and presented following the Preferred Reporting Items for Systematic Review and Meta-Analysis (PRISMA) statement [7].

### 2.1. Inclusion and Exclusion Criteria

The inclusion and exclusion criteria were determined before conducting this systematic search. We included full reports of randomized controlled trials (RCTs) investigating the pH and BE of the umbilical artery and vein and comparing phenylephrine and norepinephrine as vasopressors in parturients undergoing cesarean section.

The PICO-SD information is as follows:

- Patients (P): all parturient undergoing cesarean section undergoing spinal anesthesia.
- Intervention (I): intravenous (IV) bolus or infusion of norepinephrine.
- Comparison (C): intravenous (IV) bolus or infusion of phenylephrine.
- Outcome measurements (O): The primary outcome of this study was the umbilical artery (UA) or umbilical vein (UV) pH as the neonatal condition at birth, and the secondary outcome was umbilical artery (UA) or umbilical vein (UV) base excess (BE) as an additional prognostic value over the measurement of umbilical pH.
- Study design (SD): The full reports of randomized controlled trials (RCTs) were included. The exclusion criteria were observational studies, conference abstracts, posters, case reports, case series, comments or letters to the editor, reviews, and laboratory or animal studies.

### 2.2. Information Source and Search Strategy

Two investigators (Jee and Sung) conducted the literature search in Ovid-Medline, Ovid-EMBASE, and Cochrane Central Register of Controlled Trials (CENTRAL) databases and Google Scholar to identify all randomized controlled trials (RCTs) comparing norepinephrine and phenylephrine to prevent or treat hypotension after spinal anesthesia for cesarean section. The literature search was conducted in August 2023.

Search terms included “phenylephrine’’, “norepinephrine”, “cesarean section”, “spinal anesthesia”, and “randomized controlled trial”. The search strategies included a combination of Medical Subject Headings, EMTREE terms, and free text. Furthermore, references to the original articles included and systematic reviews in related fields were traced back to identify additional relevant articles, until no further relevant references could be found. No limitations were placed on publication date or language.

### 2.3. Study Selection

In the first stage of study selection, two investigators (Jee and Sung) independently scanned the titles and abstracts of the literature searched and excluded non-relevant literature. The works considered eligible from the first stage of study selection (assessed from the title or abstract) were subjected to the second stage of study selection. Potentially relevant studies that were identified by at least one investigator were subjected to the second stage of study selection. In addition, all abstracts that could not provide sufficient information regarding the eligibility criteria were also subjected to the second stage of study selection. In the second stage of study selection, the full paper was retrieved and evaluated. Any discrepancies for study selection were resolved through discussion. Disagreements over inclusion or exclusion were settled by discussion with a third investigator (HK).

Kappa statistics were used to measure the degree of agreement for study selection between the two independent investigators. Kappa statistics were interpreted as follows: (1) equal to 0, no agreement; (2) 0.01 to 0.20, slight agreement; (3) 0.21 to 0.40, fair agreement; (4) 0.41 to 0.60, moderate agreement; (5) 0.61 to 0.80, substantial agreement; and (6) 0.8 to 0.99, almost perfect agreement [8].

### 2.4. Data Extraction

Two independent investigators (Jee and Sung) extracted all interrelated data from the included studies and entered them into a standardized data extraction form, and then performed a cross-check. Any discrepancies were resolved through discussion. If an agreement could not be reached, the dispute was resolved with the aid of a third investigator (HK). Data extracted were as follows: (1) title, (2) name of first author, (3) name of journal, (4) year of publication, (5) study design, (6) registration of clinical trial, (7) competing interests, (8) country, (9) risk of bias, (10) inclusion criteria, (11) exclusion criteria, (12) age, (13) number of parturients, (14) twins or not and (15) primary outcome and secondary outcomes. The primary outcome of this study was the umbilical artery (UA) or umbilical vein (UV) pH as the neonatal condition at birth, and the secondary outcome was umbilical artery (UA) or umbilical vein (UV) base excess (BE) as an additional prognostic value over the measurement of umbilical pH.

We initially extracted data from tables or text. In cases involving missing or incomplete data, we tried to contact the study authors to obtain the relevant information.

### 2.5. Risk of Bias

Two independent investigators (Jee and Sung) assessed the risk of bias of the included studies using the Revised Cochrane risk of bias tool for randomized trials (RoB 2.0) version [9]. RoB 2.0 consists of five domains: (1) bias arising from the randomization process; (2) bias due to deviations from the intended interventions; (3) bias due to missing outcome data; (4) bias in the measurement of the outcome; and (5) bias in selection of the reported result. We also evaluated the overall risk of bias. It was judged as low risk when the risk of bias for all domains was low and judged as high risk when the risk of bias for at least one domain was high or the risk of biases for multiple domains were of some concern. If the overall judgement was neither low nor high, it was judged as being of some concern.

### 2.6. Data Analysis

The meta-analysis was conducted using the meta package in the R software (version 4.2.1). Two investigators (Jee and Sung) input all extracted interrelated data into the software. The weighted mean difference (MD) and their 95% confidence intervals (CIs) were calculated for each outcome. A random-effects model was used to account for clinical or methodological heterogeneity in the study. Statistical heterogeneity was assessed using an I2 test, with I2 > 50% indicating significant heterogeneity. Subgroup analysis was performed according to the outcome (umbilical artery and vein).

Meta-regression was used to identify covariates (outcome (artery vs. vein), administration method (bolus, infusion, both bolus and infusion), twin or not, and the number of parturients) that could influence the estimates (umbilical artery (UA) or umbilical vein (UV) pH and umbilical artery (UA) or umbilical vein (UV) base excess (BE)).

Publication bias was assessed using Begg’s funnel plot, Egger’s linear regression test, and Begg and Mazumdar’s rank correlation test. If the Begg’s funnel plots were visually assessed for asymmetry, or a *p* value < 0.05 was found for Egger’s linear regression test and the Begg and Mazumdar rank correlation test, publication bias was suspected.

### 2.7. Quality of the Evidence

Evidence grade was determined using the guidelines of the GRADE (Grading of Recommendations Assessment, Development and Evaluation) system, which uses a sequential assessment of the evidence quality, followed by an assessment of risk–benefit balance and a subsequent judgment on the strength of the recommendations [10].

## 3. Results

### 3.1. Search Selection

From the PubMed, EMBASE, and CENTRAL database searches, 1448 studies were initially evaluated. After adjusting for duplicates, 951 studies remained. Of these, 870 studies were excluded because they had no abstract and different designs, and 24 studies were excluded as they had no full text.

Full texts of the remaining 57 studies were reviewed in detail; 36 of these full-text studies were excluded because they were not appropriate. Twelve studies extracted from the Cochrane Library were excluded due to being ongoing studies. Only 3 [11,12,13] of 14 studies extracted from EMBASE and 18 [4,14,15,16,17,18,19,20,21,22,23,24,25,26,27,28,29] of 31 studies extracted from PubMed were eligible. The excluded papers were excluded because of being review articles or abstracts of conference presentations, different study designs, a lack of umbilical cord blood analysis or a lack of calculation of CI through statistical processing. Detailed descriptions for excluded studies are presented in Supplementary Materials.

**Figure 1:**
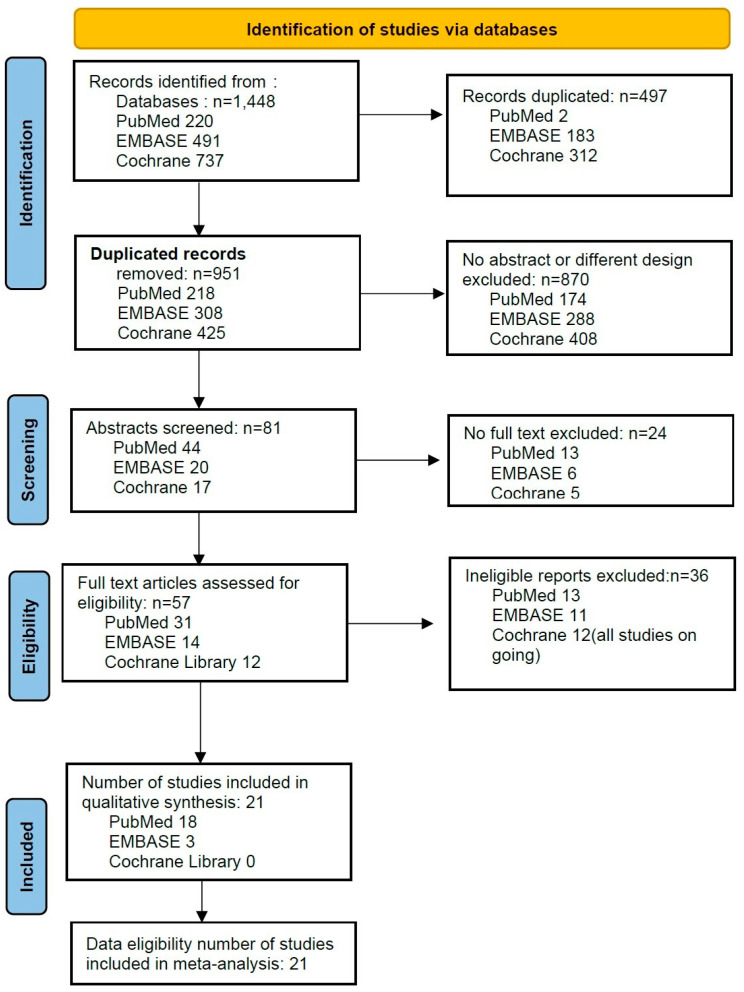
Flowchart study of the selection.

Thus, twenty-one studies including a total of 1628 patients were included in this study (Figure 1, Table 1).

### 3.2. Description of Trials

The characteristics of the 21 studies, in accordance with the rigorous inclusion criteria, are described in Table 1. Of the 21 studies, 18 studies were extracted from PubMed and 3 studies from EMBASE.

### 3.3. Umbilical Artery (UA) or Umbilical Vein (UV) pH

**Figure 2:**
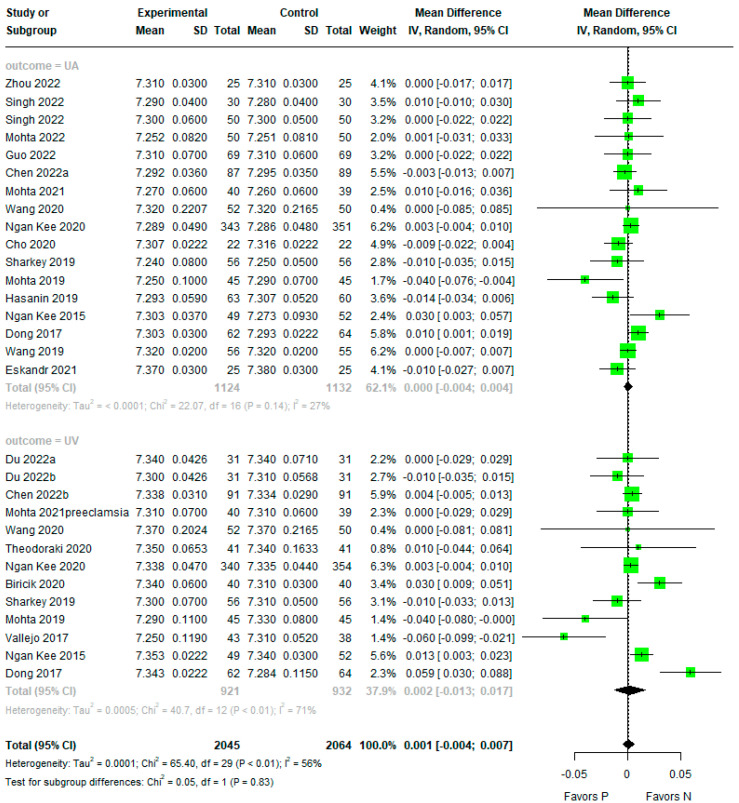
Forest plot for umbilical artery (UA) or umbilical vein (UV) pH. The figure depicts individual trials as filled green circles, with relative sample size and the 95% confidence interval (CI) of the difference as a solid line. The diamond shape indicates the pooled estimate and uncertainty regarding the combined effect [4,11,12,13,14,15,16,17,18,19,20,21,22,23,24,25,26,27,28,29,30].

There was no evidence of a difference between phenylephrine and norepinephrine for overall pH (mean difference (MD) −0.001, 95% confidence interval (CI) −0.004 to 0.007, Tau2 = 0.0001, Pchi2 < 0.01, I2 = 58%) (Figure 2). The subgroup analysis also showed no evidence of differences for UA and UV pH (MD 0.000, 95%CI −0.004 to 0.004, Tau2 ≤ 0.0001, Pchi2 = 0.14, I2 = 27% and MD 0.002, 95%CI −0.013 to 0.017, Tau2 = 0.0005, Pchi2 < 0.01, I2 = 71%).

**Figure 3:**
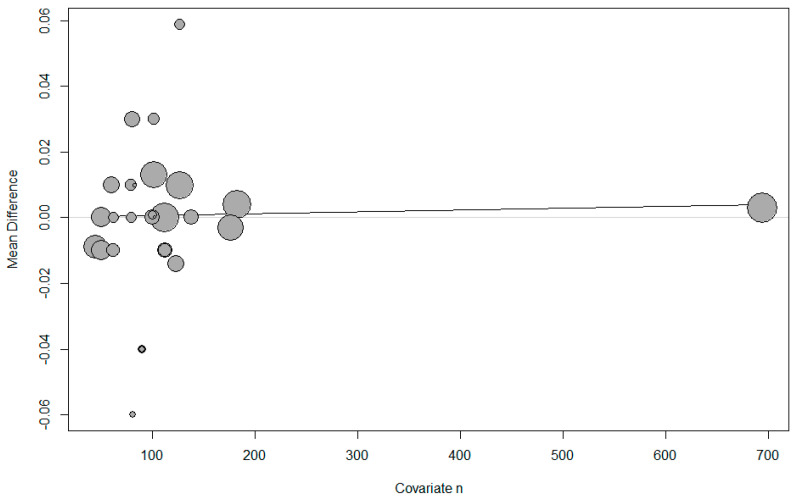
Meta-regression of umbilical pH by number of parturients. The x-axis represents the number of parturients and the y-axis represents mean difference in pH. The size of the data marker is proportional to the weight in the meta-regression.

Meta-regression showed that the use of the umbilical artery or vein, the infusion method, whether single or twin pregnancy, and the number of parturients per study had no effect on the umbilical pH (Table 2, Figure 3).

**Figure 4:**
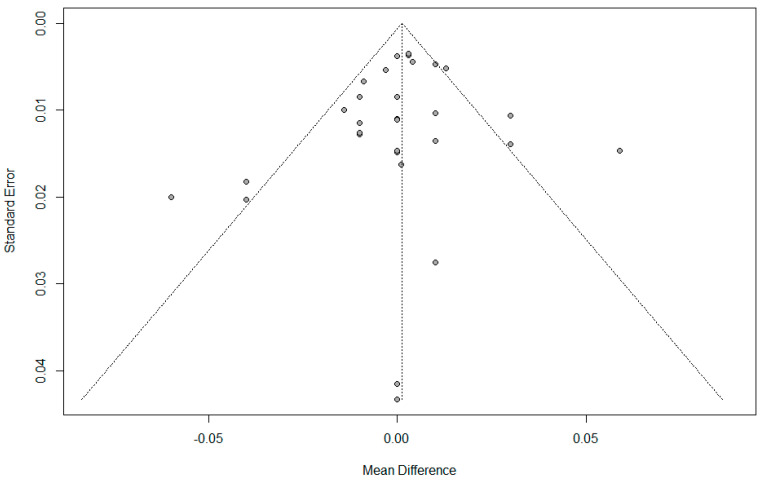
Funnel plot for umbilical pH.

There was no evidence of publication bias detected by Begg’s funnel plot, Egger’s linear regression test (*p* = 0.399), or Begg and Mazumdar’s rank correlation test (*p* = 0.748). (Figure 4).

### 3.4. Base Excess

**Figure 5:**
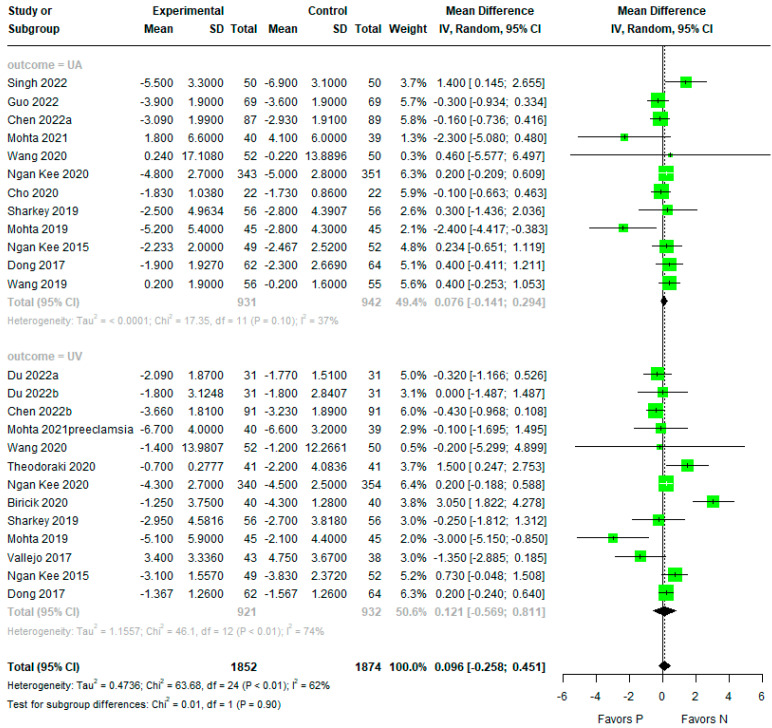
Forest plot for umbilical artery (UA) or umbilical vein (UV) BE. The figure depicts individual trials as filled green circles, with relative sample size and the 95% confidence interval (CI) of the difference as a solid line. The diamond shape indicates the pooled estimate and uncertainty for the combined effect [4,13,15,16,17,19,20,22,23,25,26,27,28,29,30].

There was no evidence of any difference between phenylephrine and norepinephrine for overall BE (MD 0.096, 95% CI −0.258 to 0.451, Tau2 = 0.4736, Pchi2 < 0.01, I2 = 62%) (Figure 5). The subgroup analysis also showed no evidence of differences for UA and UV BE (MD 0.076, 95% CI −0.141 to 0.294, Tau2 =< 0.0001, Pchi2 = 0.10, I2 = 37% and MD 0.121, 95% CI; −0.569 to 0.811, Tau2 = 1.1557, Pchi2 < 0.01, I2 = 74%).

**Figure 6:**
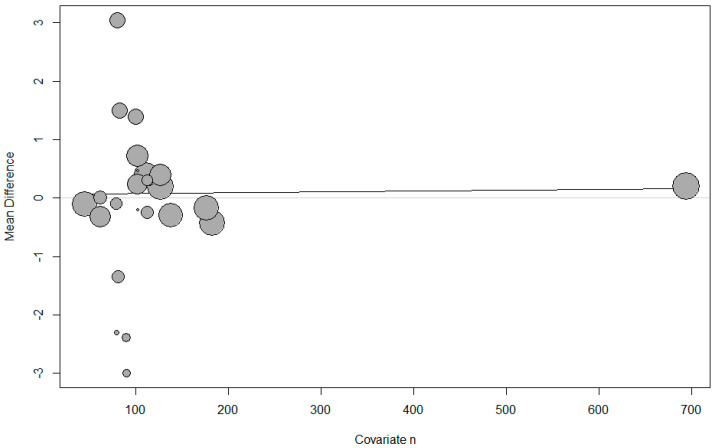
Meta-regression of umbilical base excess by number of parturients. The s-axis represents the number of parturients and the y-axis represents the mean difference in base excess. The size of the data marker is proportional to the weight in the meta-regression.

The meta-regression showed that umbilical artery or vein, infusion method, single or twin pregnancy, and the number of parturients per study had no effect on the umbilical BE (Table 2, Figure 6).

**Figure 7:**
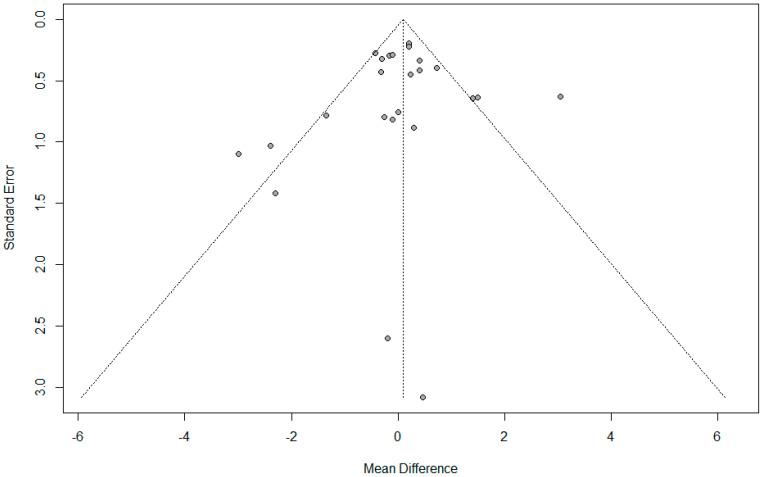
Funnel plot for umbilical base excess.

There was no evidence of publication bias detected by the Begg’s funnel plot, Egger’s linear regression test (*p* = 0.650), or Begg and Mazumdar’s rank correlation test (*p* = 0.243) (Figure 7).

### 3.5. Risk of Bias

**Figure 8:**
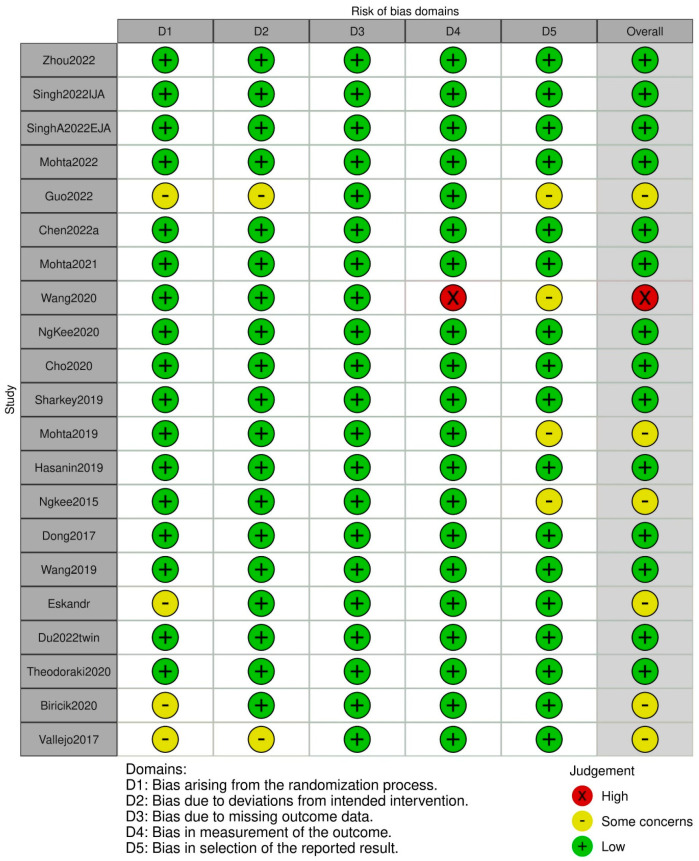
The risk of bias assessment [4,11,12,13,14,15,16,17,18,19,20,21,22,23,24,25,26,27,28,29,30].

The risk of bias assessment performed using the Cochrane tool for the included studies is presented in Figure 8. Among the twenty-one included studies, bias arising from the randomization process, bias due to deviations from the intended interventions, and bias in the selection of the reported result were assessed as presenting “some concerns” in four studies, two studies, and four studies, respectively, and bias in the measurement of the outcome was assessed as “high risk” in one study. Consequently, the overall risk of bias was assessed as being of “some concern” in seven studies, and as “high risk” in one study.

### 3.6. GRADE

Four outcomes were evaluated using the GRADE system (Table 3). The qualities of UA pH and UA BE were evaluated as “high’, and the qualities of overall PH, overall BE, UV pH, and UV BE were evaluated as “moderate’.

## 4. Discussion

The results of the current systematic review and meta-analysis showed that there was no evidence of differences between phenylephrine and norepinephrine for overall pH and BE. The subgroup analysis also showed no evidence of differences for UA and UV regarding pH and BE. The meta-regression showed that differentiating between umbilical artery or vein, infusion method, single or twin pregnancy, and the number of parturients per study did not affect umbilical pH and BE.

This finding suggests that norepinephrine demonstrates comparable fetal safety to phenylephrine. The study specifically examined the potential adverse impact of norepinephrine on the fetus, focusing on objective measures such as umbilical cord blood pH and BE, while excluding subjective indicators like the APGAR score. The choice of UA, UV, or both varied across studies, and despite heterogeneity in the sample, all data values were considered regardless of injection method (bolus or infusion), normal-health mothers with multiple pregnancies or singleton pregnancies, or mothers with gestational hypertension, due to the limited existing research on the topic.

In light of the observed ease of breakdown in the placenta and the minimal placental transfer, it is not unexpected that norepinephrine may have a negligible effect on the fetus [31]. The influence of norepinephrine on the fetal acid–base balance appears to be positive, similar to phenylephrine, with no apparent adverse effects on the fetus. Based on the results of this study, the application of norepinephrine to prevent hypotension in cesarean section can be considered as safe for fetal well-being as phenylephrine.

Post-spinal hypotension has been reported to occur in between 7.4% and 74.1% of mothers during cesarean section, and the use of vasopressors is the most effective method in the prevention of hypotension [32]. A couple of decades ago, ephedrine was the preferred drug in obstetric anesthesia due to its ability to maintain uterine blood flow. Phenylephrine, on the other hand, was initially avoided due to concerns about its potential adverse effects on uterine blood flow [1]. Subsequent evidence demonstrating the superior advantages of phenylephrine over ephedrine for fetal acid–base balance has led to a widespread consensus favoring phenylephrine as the drug of choice for preventing post-spinal hypotension during cesarean sections [2].

Phenylephrine, a selective α1 antagonist, induces arteriolar vasoconstriction, resulting in increased arterial pressure and a baroreceptor-triggered, vagally mediated reduction in heart rate and cardiac output. However, the drawback of phenylephrine lies in its tendency to cause severe bradycardia and decreased cardiac output. Attempts to address this issue through the prescription of the anticholinergic glycopyrrolate proved to be temporary and insufficient to solve this problem [3].

To address the persistent problem of bradycardia and decreased cardiac output associated with phenylephrine, Ngan Kee conducted a double-blinded, randomized clinical trial comparing norepinephrine and phenylephrine infusion for maintaining blood pressure during cesarean section under spinal anesthesia [4]. The study revealed that the continuous infusion of norepinephrine effectively maintained blood pressure, comparable to phenylephrine, but with fewer instances of bradycardia and a lower reduction in cardiac output. Neonatal outcomes did not significantly differ between the two drugs. The authors proposed that the favorable maintenance of blood pressure with norepinephrine could be attributed to its β-adrenergic receptor agonist activity in addition to its α-adrenergic receptor effects.

Nevertheless, obstetric anesthesiologists have expressed their concern regarding the use of norepinephrine in women undergoing cesarean sections due to unresolved concerns related to maternal safety. Issues such as tissue injury resulting from norepinephrine extravasation and local vasoconstriction remain unsettled, raising concerns about the practical clinical application of norepinephrine in this context. Ngan Kee’s assertion that a concentration of 50 mcg/mL norepinephrine, equating to the potency of 80 mcg/mL phenylephrine, carries no risk of inducing tissue injury provides a reassuring perspective [33].

In addition, the manufacturer of norepinephrine recommends administering norepinephrine through a large vein, such as the antecubital vein, while avoiding peripheral blood vessels in the lower extremities [34]. A central venous line is not required with this approach. Consequently, it is believed that the risk of tissue injury resulting from norepinephrine extravasation and local vasoconstriction in pregnant women is minimized when norepinephrine is appropriately diluted and injected into a large blood vessel. The utilization of norepinephrine in obstetric anesthesia differs from its use in the intensive care unit (ICU) as it is more diluted and deemed safer when rapidly administered with fluid co-loading. Typically, it is employed in mothers who have undergone regional anesthesia, such as spinal anesthesia, facilitating easy detection while the mother is awake.

In light of these considerations, it appears that the widespread acceptance of norepinephrine in obstetric anesthesia for preventing hypotension after spinal anesthesia during cesarean sections is imminent.

Our study has several limitations. Firstly, the results of the systematic review and meta-analysis revealed substantial heterogeneity due to diverse protocols, infusion methods, and varying doses of phenylephrine and norepinephrine in the included studies. Additionally, there were variations in umbilical artery and vein selection, as well as the number of parturients, which contributed to considerable heterogeneity. To address this, we performed a subgroup analysis by dividing studies based on the umbilical artery and vein. We also conducted a meta-regression to identify covariates (e.g., outcome, administration method, twin status, and number of parturients) that could influence estimates (umbilical artery (UA) or umbilical vein (UV) pH and base excess (BE)).

Secondly, only the published trials were included in this meta-analysis. Nonetheless, our current meta-analysis is a systematic review encompassing the maximum number of trials to compare the pH and BE of the umbilical artery and vein in parturients undergoing cesarean section when treated with phenylephrine or norepinephrine. Thirdly, even after comprehensive and sensitive searching, only twenty-one studies with a total of 1628 patients were included in this study. For some outcomes, it may have been underpowered; therefore, the findings from the study are inconclusive.

Despite these limitations, our study exhibited strength through its rigorous methodology in systematically reviewing and meta-analyzing pH and BE differences in the umbilical artery and vein between phenylephrine and norepinephrine in parturients undergoing cesarean sections.

In conclusion, no evidence of differences between phenylephrine and norepinephrine was found for overall pH and BE. The subgroup analysis revealed no evidence of differences for UA and UV, and the meta-regression indicated that factors such as umbilical artery or vein, infusion method, twin status, and the number of parturients per study did not significantly affect umbilical pH and BE.

## Figures and Tables

**Table 1:** Characteristics of studies.

First Author, Year, Country	Participants	Anesthesia	Mode of Administration	Intervention	Outcome
Zhou, 2022, China [14]	50 healthy women	CSEA with hyperbaric 0.5% bupivacaine 12.5 mg	bolus + continuous infusion	Bolus of NE 4 ug vs. PE 50 ug + continuous infusion of (NE 8 μg/mL vs. PE 100 ug/mL) at a rate of 30 mL/h.	primary outcome: neonatal UA/ UV BGA, APGAR score (1, 5 min); secondary outcome: maternal SBP, HR, IONV
Singh, 2022, India [17]	100 healthy women	SA with 0.5% hyperbaric bupivacaine 10 mg + fentanyl 25 μg	continuous infusion	PE 100 ug/min vs. NE 5 μg/min to maintain SBP 90–110% baseline.	primary outcome: neonatal UA/ UV BGA, APGAR score (1, 5 min); secondary outcome: maternal SBP, HR, IONV
Singh, 2022, India [18]	60 healthy women	SA with 0.5% bupivacaine 11 mg		Continuous infusion of PE 50 μg/min vs. NE 2.5 μg/min.	
Mohta, 2022, India [21]	100 healthy women	SA with 0.5% hyperbaric bupivacaine 10–11 mg	intermittent bolus	Bolus of PE 100 μg or NE 8 μg when SBP <100 mmHg.	primary outcome: neonatal UA/UV BGA, APGAR score; secondary outcome: rescues of vasopressor, episodes of hypotension, incidence of bradycardia/tachycardia/arrhythmias, IONV
Guo, 2022, China [25]	138 pre-eclampsia women	SA with 0.5% hyperbaric bupivacaine 12.5 mg	continuous infusion	Continuous infusions of PE 0.625 μg /kg/min vs. NE 0.05 μg/kg/min.	primary outcome: incidence of bradycardia; secondary outcome: incidence of hypotension, hypertension, IONV, stability of HR, sbp, UA BGA, APGAR score
Du, 2022, China [26]	62 twin pregnancies	SA with 0.5% isobaric ropivacaine 12 mg + sufentanil 5 μg	continuous infusion	Continuous infusion of NE 6 μg/min vs. phenylepinephrine 75 μg/min.	primary outcome: maternal episodes of hypotension, bradycardia, reactive hypertension, N/V; secondary outcome: neonatal APGAR (1, 5 min), UV BGA
Chen, 2022, China [28]	100 healthy women	SA with of 0.5% isobaric bupivacaine 12.5 mg	continuous infusion	Continuous infusion of NE 3.2 μg/min or PE infusion 40 μg/min. Bolus of NE 8 μg vs. PE 100 μg for parturients with PE when SBP < 90 mmHg or 80% baseline.	primary outcome: maternal SBP, HR; secondary outcome: neonatal APGAR score, UA UV BGA.
Mohta, 2021, India [22]	86 pre-eclampsia, singleton	SA with hyperbaric 0.5% bupivacaine 11 mg	intermittent bolus	Bolus of PE 50 μg or NE 4 μg when SBP <100 mmHg, SBP fall 20% from the baseline.	primary outcome: umbilical artery pH; secondary outcomes: APGAR scores (1 and 5 min), the number of hypotensive episodes, vasopressor boluses, tachycardia, bradycardia, arrhythmias or hypertension and maternal complications.
Wang, 2020, China [30]	102 healthy women	SA with 0.5% ropivacaine 15 mg	intermittent bolus	Bolus of NE 8 µg vs. PE 100 µg when SBP <80%.	primary outcome: maternal SBP, HR, CO, SV, TPR; secondary outcomes: neonatal APGAR, UA/UV BGA
Theodoraki, 2020, Greece [16]	82 healthy women	CSEA with 0.75% ropivacaine 13.5 mg + fentanyl 10 μg.	continuous infusion	Continuous infusion rate of PE 50 μg/min vs. NE 4 μg/min.	primary outcome: maternal bradycardia episodes, incidences of hypotension, hypertension; secondary outcome: neonatal UV BGA, APGAR score < 7
Ngan Kee, 2020, China [20]	668 women elective/non-elective CS under spinal or CSE anesthesia	SA or CSEA with hyperbaric bupivacaine 0.5% + fentanyl with no restriction on dose.	intravenous infusion or intermittent boluses, or both according to individual preference.	NE 6 μg/mL or PE 100 μg/mL either prophylactically or therapeutically, as an infusion or bolus.	primary outcome: neonatal APGAR score < 7, UA/UV BGA
Cho, 2020, South Korea [27]	44 healthy women	SA with hyperbaric bupivacaine 8 mg with fentanyl 15 μg	intermittent bolus	Bolus of study drug whenever hypotension occurred. Hypotension was defined as < 80% baseline SBP or < 90 mmHg.	primary outcome: maternal outcome (SBP, HR, CO, SV, SVR); secondary outcome: neonatal outcomes (APGAR scores 1, 5 min; UA BGA)
Biricik, 2020, Turkey [29]	80/160 healthy women	SA with hyperbaric bupivacaine 10 mg + fentanyl 20 μg	continuous infusion	5 μg/mL NE, 100 μg/mL infused at a 30 mL/h.	primary outcome: APGAR, UA pH (IQR); secondary outcome: maternal hypotension incidence, number of patients receiving ephedrine rescue, mean ephedrine consumption
Sharkey, 2019, Canada [19]	112 healthy women	SA with 0.75% hyperbaric bupivacaine 13.5 mg + fentanyl 10 μg + morphine 100 μg	intermittent bolus	Bolus of 100 μg/mL PE vs. 6 μg/mL NE if SBP lower baseline 80% + HR <60 bpm or if SBP < 80% of baseline.	primary outcome: maternal bradycardia (HR <50 bpm); secondary outcomes: incidences of maternal bradycardia; hypotension, hypertension, tachycardia, IONV; block level; UC and UV BGs; APGAR scores
Mohta, 2019, India [23]	90 healthy women	SA with hyperbaric 0.5% bupivacaine 10–11 mg,	intermittent bolus	Bolus of PE 100 μg/mL vs. NE 5 μg/mL when SBP < 100 mmHg or below 20% of baseline.	primary outcome measure: maternal bradycardia; secondary outcome measures included changes in maternal systolic arterial pressure after vasopressor administration; number of episodes of hypotension and reactive hypertension; number of vasopressor doses used to treat first hypotensive episode and the total number required until delivery of baby; incidence of maternal complications, for example, nausea, vomiting, dizziness; APGAR scores at 1 min and 5 min; umbilical artery pH; and incidence of fetal acidosis, defined as umbilical artery pH < 7.20.
Hasanin, 2019, Egypt [24]	123 healthy women	SA with hyperbaric bupivacaine 10 mg + fentanyl 20 μg	continuous infusion	NE infusion rate 0.05 μg/kg/min diluted 4 μg/mL; PE infusion ratem0.75 μg/kg/min diluted 50 μg/mL.	primary outcome: maternal hemodynamic parameter; secondary outcome: neonatal UA blood gas, APGAR
Vallejo, 2017, USA [15]	81 healthy women	SA with hyperbaric bupivacaine 12–15 mg + morphine 0.2 mg + fentanyl 20 μg	continuous infusion	Continuous infusion of PE 0.1 μg/kg/min vs. NE 0.05 μg/kg/min for SBP within 100–120% of baseline.	maternal outcome: SBP, DBP, HR, CO, CI, SV, SVR, vasopressor rescues; neonatal outcome: APGAR score, UV BGA
Ngan Kee, 2015, China [4]	101 healthy women	SA with 0.5% hyperbaric bupivacaine 11 mg + fentanyl 15 μg	continuous infusion	Computer-controlled infusion of NE 5 μg/mL vs. PE 100 μg/mL.	primary outcome: maternal SBP, HR, SVR, SV, CO; secondary outcome: neonatal APGAR, UA/UV BGA
Dong, 2017, China [13]	126 healthy women	SA with 0.5% ropivacaine 15 mg	intermittent bolus	Bolus of NE 10 μg (10 μg/mL) vs. PE 50 μg (50 μg/mL).	primary outcome: neonatal APGAR, UA/UV blood gas; secondary outcome: incidence of maternal hypertension, bradycardia, no. of vasopressor rescues
Wang, 2019, China [11]	111/166 pre-eclampsia parturients	SA with 0.5% bupivacaine 10–11 mg	intermittent bolus	Bolus of NE 4 μg vs. PE 50 μg.	primary outcome: maternal SBP, HR; secondary outcome: incidence of tachycardia, bradycardia, hypertension, no. of vasopressor rescues, APGAR, UA blood gas, pH
Eskandr, 2021, Egypt [12]	50/75 healthy women	SA with 0.5% hyperbaric bupivacaine 9–13 mg + fentanyl 25 μg	intermittent bolus	Bolus of PE 0.2 μg/kg vs. NE 0.1 μg/kg.	APGAR, umbilical blood pH, acidosis

CSEA: combined spinal epidural anesthesia, NE: norepinephrine, PE: phenylephrine, UA: uterine artery, UV: uterine vein, BGA: blood gas analysis, SBP: systolic blood pressure, HR: heart rate, IONV: intraoperative nausea and vomiting, SA: spinal anesthesia, MBP: mean blood pressure, CO: cardiac output, SV: stroke volume, DBP: diastolic blood pressure, CI: cardiac index, SVR: systemic vascular resistance.

**Table 2:** Results from the meta-regression analysis.

	pH		BE	
	*p*-Value		*p*-Value	
UA or UV	0.3921	UApH −0.0006 (−0.0072 to 0.0061)		
		UVpH 0.0042 (−0.0044 to 0.0127)		
Infusion method	0.9291	Infusion 0.0016 (−0.0066; 0.0098)	0.3293	Infusion 0.3444 [−0.2158; 0.9045]
		bolus −0.0004 (−0.0102; 0.0094)		Bolus −0.2956 [−0.9356; 0.3443]
		Infusion or bolus 0.0030 [−0.0145; 0.0205]		Infusion or bolus 0.2000 [−0.9450; 1.3450]
Single or twin	0.7037	Single 0.0016 [−0.0045; 0.0077]	0.3105	Single 0.1887 [−0.1965; 0.5739]
		Twin −0.0013 [−0.0153; 0.0126]		Twin −0.2570 [−1.0273; 0.5134]
Number of parturients	0.700	Number of patients 0.000 (−0.000 to 0.000)	0.881	Number of patients 0.000 (−0.002 to 0.002)

UA: uterine artery, UV: uterine vein, BE: base excess.

**Table 3:** The GRADE evidence quality for each outcome.

Outcomes	Number of Studies	Quality Assessment					Heterogeneity		MD (95% CI)	Quality
		ROB	Inconsistency	Indirectness	Imprecision	Publication Bias	Tau(τ)^2^	I^2^		
pH overall	24	not serious	serious	not serious	not serious	not serious	0.0001	I^2^ = 58%	−0.001 (−0.004 to 0.007)	⨁⨁⨁○ Moderate
pH UA	17	not serious	not serious	not serious	not serious	not serious	< 0.0001	27%	0.000 (−0.004 to 0.004)	⨁⨁⨁⨁ High
pH UV	13	not serious	serious	not serious	not serious	not serious	0.0005	71%	0.002 (−0.013 to 0.017)	⨁⨁⨁○ Moderate
BE overall	17	not serious	serious	not serious	not serious	not serious	0.4736	62%	0.096 (−0.258 to 0.451)	⨁⨁⨁○ Moderate
pH BE	41	not serious	not serious	not serious	not serious	not serious	0.0001	37%	0.076 (−0.141 to 0.294)	⨁⨁⨁⨁ High
pH U BE		not serious	serious	not serious	not serious	not serious	1.1557	74%	0.121 (−0.569 to 0.811)	⨁⨁⨁○ Moderate

A: artery, V: vein, CI: confidence interval, ROB: risk of bias, MD: mean difference, NA. GRADE Working Group grades of evidence: ⨁⨁⨁⨁ High quality: We are very confident that the true effect lies close to the estimated effect. ⨁⨁⨁○ Moderate quality: We are moderately confident in the effect estimate; the true effect is likely to be close to the estimate of the effect, but there is a possibility that it is substantially different.

## Data Availability

The datasets generated and/or analyzed in the current study are available from the corresponding author upon request.

## Supplementary Materials

The following supporting information can be downloaded at: https://www.mdpi.com/article/10.3390/jpm14080803/s1, The excluded studies.
