# Supplementary material for: A Comparison of Norepinephrine versus Phenylephrine to Prevent Hypotension after Spinal Anesthesia for Cesarean Section: Systematic Review and Meta-Analysis
Source: J Pers Med. 2024 Jul 29;14(8):803. doi: 10.3390/jpm14080803 (PMC11355940; doi:10.3390/jpm14080803)
Supplement: Supplementary file 1 [file jpm-14-00803-s001.zip › jpm-3096662-supplementary.pdf]

Supplementary Materials

The excluded studies

Twelve studies extracted from Cochrane Library

https://clinicaltrials.gov/show/NCT05035485 2021,

https://clinicaltrials.gov/show/NCT05035498 2021

https://clinicaltrials.gov/show/NCT05035888 2021

https://clinicaltrials.gov/show/NCT05035472 2021

https://clinicaltrials.gov/show/NCT03421860 2018

https://clinicaltrials.gov/show/NCT03702400 2018

https://clinicaltrials.gov/show/NCT03328533 2017

https://clinicaltrials.gov/show/NCT03248791 2017

https://clinicaltrials.gov/show/NCT02854787 2016

https://clinicaltrials.gov/show/NCT02969239 2016

https://clinicaltrials.gov/show/NCT03015857 2016

https://clinicaltrials.gov/show/NCT02354833 2015

Eleven studies excluded from EMBASE

1. M. C. Vallejo, O. M. Elzamzamy, D. T. Cifarelli, A. L. Phelps, A. Attaallah, P. Ranganathan, et al. Comparison of continuous iv phenylephrine vs. Norepinephrine infusion in prevention of spinal hypotension during caesarean delivery: Assessment of hemodynamic parameters and maternal outcomes. Anesthesia and Analgesia 2016

Vol. 122 Issue 5 Pages S186

2. S. G. Osmani, M. Acharya, S. S. Kamath, Y. V. Suresh and K. Prabhu. Comparison of prophylactic phenylephrine versus noradrenaline boluses for hemodynamic stability during elective cesarean delivery under spinal anesthesia-an observational study. Anaesthesia, Pain and Intensive Care 2022 Vol. 26 Issue 2 Pages 168-174.

3. T. Muthiah, R. Banupriya, S. Rajeshwari and A. Chhabra. Prophylactic infusion of phenylephrine vs. norepinephrine for the prevention of post spinal hypotension in parturients undergoing elective caesarean section - A randomised control trial. Anaesthesia 2019 Vol. 74 Pages 18.

4. M. I. Mehdi, S. Arya, P. Verma and S. Singh. Comparative Assessment of the Use of Norepinephrine with Phenylephrine During Spinal Anaesthesia for Caesarean Birth. International Journal of Pharmaceutical and Clinical Research 2021 Vol. 13 Issue 4 Pages 389-395.

5. F. Khatoon, R. Fernando, A. Naz, F. Khalid, E. Abdulla, M. Columb, et al. Relative potency of noradrenaline vs. phenylephrine infusions in the prevention of hypotension after spinal anaesthesia for caesarean delivery. International Journal of Obstetric Anesthesia 2019 Vol. 39 Pages 8-7.

6. W. N. Kee, F. F. Ng, S. W. Y. Lee and A. Lee. Norepinephrine versus phenylephrine during spinal anaesthesia for caesarean delivery: a randomized double-blinded pragmatic non-inferiority study comparing neonatal outcome.

International Journal of Obstetric Anesthesia 2019 Vol. 39 Pages 7.

7. A. Iqbal, H. M. Javed, K. Ahmad, M. A. Ashfaq, M. R. Butt and M. Khalid. Phenylephrine Boluses Vs Noradrenaline Boluses for Hypotension after Spinal Anesthesia in LSCS. Pakistan Journal of Medical and Health Sciences 2022 Vol. 16 Issue 7 Pages 775-777.

8. E. Biricik, F. Karacaer, İ. Ünal, M. Sucu and H. Ünlügenç. The effect of epinephrine for the treatment of spinal-hypotension: comparison with norepinephrine and phenylephrine, clinical trial. Brazilian Journal of Anesthesiology 2020 Vol. 70 Issue 5 Pages 500-507.

9. E. Biricik, F. Karacaer, I. Ünal, M. Sucu and H. Ünlügenç. The effect of epinephrine for the treatment of spinal-hypotension: Comparison with norepinephrine and phenylephrine. Regional Anesthesia and Pain Medicine 2019 Vol. 44 Issue 10 Pages A80.

10. C. V. S. Ali. Comparative study of prophylactic infusions of phenylephrine and norepinephrine for the management of maternal hypotension for cesarean section under spinal anesthesia. Indian Journal of Critical Care Medicine 2021 Vol. 25 Issue SUPPL 1 Pages S91-S92.

11. M. Mohta, A. Garg and G. T. Chilkoti. Randomised double-blind comparison of phenylephrine and norepinephrine boluses for treatment of post-spinal hypotension during elective caesarean section. Regional Anesthesia and Pain Medicine 2018 Vol. 43 Issue 7 Pages e44-e45.

Thirteen studies excluded from PubMed.

1. J. P. Tiwari, S. J. Verma and A. K. Singh. A Prospective Randomized Study Comparing the Bolus Doses of Norepinephrine and Phenylephrine for the Treatment of Spinal Induced Hypotension in Cesarean Section. Cureus 2022 Vol. 14 Issue 7 Pages e27166.

2. N. Puthenveettil, S. N. Sivachalam, S. Rajan, J. Paul and L. Kumar. Comparison of norepinephrine and phenylephrine boluses for the treatment of hypotension during spinal anaesthesia for caesarean section - A randomised controlled trial. Indian J Anaesth 2019 Vol. 63 Issue 12 Pages 995-1000.

3. W. D. Ngan Kee, K. S. Khaw, Y. H. Tam, F. F. Ng and S. W. Lee. Performance of a closed-loop feedback computer-controlled infusion system for maintaining blood pressure during spinal anaesthesia for caesarean section: a randomized controlled comparison of norepinephrine versus phenylephrine. J Clin Monit Comput 2017 Vol. 31 Issue 3 Pages 617-623.

4. W. D. Ngan Kee. A Random-allocation Graded Dose-Response Study of Norepinephrine and Phenylephrine for Treating Hypotension during Spinal Anesthesia for Cesarean Delivery. Anesthesiology 2017 Vol. 127 Issue 6 Pages 934-941.

5. M. Mostafa, A. Hasanin, M. Mostafa, M. Y. Taha, M. Elsayad, F. A. Haggag, et al. Hemodynamic effects of norepinephrine versus phenylephrine infusion for prophylaxis against spinal anesthesia-induced hypotension in the elderly population undergoing hip fracture surgery: a randomized controlled trial. Korean J Anesthesiol

2021 Vol. 74 Issue 4 Pages 308-316.

6. M. Mohta, M. Dubey, R. K. Malhotra and A. Tyagi. Comparison of the potency of phenylephrine and norepinephrine bolus doses used to treat post-spinal hypotension during elective caesarean section. Int J Obstet Anesth 2019 Vol. 38 Pages 25-31.

7. K. Goel, N. Luthra, N. Goyal, A. Grewal and A. Taneja. Comparison of norepinephrine and phenylephrine infusions for maintenance of haemodynamics following subarachnoid block in lower segment caeserean section. Indian J Anaesth 2021 Vol. 65 Issue 8 Pages 600-605.

8. K. Feng, X. Wang, X. Feng, J. Zhang, W. Xiao, F. Wang, et al. Effects of continuous infusion of phenylephrine vs. norepinephrine on parturients and fetuses under LiDCOrapid monitoring: a randomized, double-blind, placebo-controlled study. BMC Anesthesiol 2020 Vol. 20 Issue 1 Pages 229.

9. X. Wang, X. Shen, S. Liu, J. Yang and S. Xu. The Efficacy and Safety of Norepinephrine and Its Feasibility as a Replacement for Phenylephrine to Manage Maternal Hypotension during Elective Cesarean Delivery under Spinal Anesthesia. Biomed Res Int 2018 Vol. 2018 Pages 1869189.

10. S. Xu, X. Shen, S. Liu, J. Yang and X. Wang. Efficacy and safety of norepinephrine versus phenylephrine for the management of maternal hypotension during cesarean delivery with spinal anesthesia: A systematic review and meta-analysis. Medicine (Baltimore) 2019 Vol. 98 Issue 5 Pages e14331.

11. J. Qian, Y. P. Zhao, J. L. Deng, L. Z. Wang, F. Xiao, B. Shen, et al. Determination of the Relative Potency of Norepinephrine and Phenylephrine Given as Infusions for Preventing Hypotension During Combined Spinal-Epidural Anesthesia for Cesarean Delivery: A Randomized Up-And-Down Sequential Allocation Study. Front Pharmacol 2022 Vol. 13 Pages 942005.

12. M. Heesen, N. Hilber, K. Rijs, R. Rossaint, T. Girard, F. J. Mercier, et al. A systematic review of phenylephrine vs. noradrenaline for the management of hypotension associated with neuraxial anaesthesia in women undergoing caesarean section. Anaesthesia 2020 Vol. 75 Issue 6 Pages 800-808.

13. Y. Wei and X. Zheng. A commentary on "Prevention of hypotension during elective cesarean section with a fixed-rate norepinephrine infusion versus a fixed-rate phenylephrine infusion. A double-blinded randomized controlled trial" (Int J Surg 2020; 84:41-49)
